# TALEN mediated targeted editing of GM2/GD2-synthase gene modulates anchorage independent growth by reducing anoikis resistance in mouse tumor cells

**DOI:** 10.1038/srep09048

**Published:** 2015-03-12

**Authors:** Barun Mahata, Avisek Banerjee, Manjari Kundu, Uday Bandyopadhyay, Kaushik Biswas

**Affiliations:** 1 Division of Molecular Medicine, Bose Institute, Kolkata, India; 2 Department of Infectious Diseases and Immunology, CSIR-Indian Institute of Chemical Biology, Kolkata, India

## Abstract

Complex ganglioside expression is highly deregulated in several tumors which is further dependent on specific ganglioside synthase genes. Here, we designed and constructed a pair of highly specific transcription-activator like effector endonuclease (TALENs) to disrupt a particular genomic locus of mouse GM2-synthase, a region conserved in coding sequence of all four transcript variants of mouse GM2-synthase. Our designed TALENs effectively work in different mouse cell lines and TALEN induced mutation rate is over 45%. Clonal selection strategy is undertaken to generate stable GM2-synthase knockout cell line. We have also demonstrated non-homologous end joining (NHEJ) mediated integration of neomycin cassette into the TALEN targeted GM2-synthase locus. Functionally, clonally selected GM2-synthase knockout clones show reduced anchorage-independent growth (AIG), reduction in tumor growth and higher cellular adhesion as compared to wild type Renca-v cells. Insight into the mechanism shows that, reduced AIG is due to loss in anoikis resistance, as both knockout clones show increased sensitivity to detachment induced apoptosis. Therefore, TALEN mediated precise genome editing at GM2-synthase locus not only helps us in understanding the function of GM2-synthase gene and complex gangliosides in tumorigenicity but also holds tremendous potential to use TALENs in translational cancer research and therapeutics.

Gangliosides are sialic acid containing glycosphingolipids, ubiquitous in mammalian cells and predominant in the outer leaflet of the lipid bilayer of the cell membrane. They play multiple roles acting as cell surface receptor and markers, participating in intercellular communication and modulating cell signaling, cell cycle and cellular motility^1^,^2^. During the past few years, gangliosides have emerged as one of the major players in mediating tumor-induced immune suppression. Several of these gangliosides are not only found to be over-expressed in various tumors but also actively shed from tumor cell surface into the surrounding tumor microenvironment, thereby modulating host immune response^3^,^4^,^5^. Gangliosides shed in the tumor microenvironment possess potent immune-suppressive properties which interfere and block an effective anti-tumor immune response. Tumor-derived gangliosides (GM1, GM2, GD3) have already been documented to cause immune cell dysfunction through their ability to kill T cells by apoptosis or by impairing antigen presentation by dendritic cells^6^,^7^,^8^,^9^. Apart from their deleterious role on immune cells, studies have shown complex roles of these gangliosides on tumor cell behavior as well. For example, ganglioside GM3 was found to be anti-angiogenic in malignant brain tumor^10^. Interestingly, neo-synthesis of complex gangliosides (GM2 and a-series) increased the mitotic index and vascular density through the enhanced expression of VEGF *in vivo*, thereby contributing to increased angiogenesis^11^. Though data from previous studies have demonstrated the ability of GM3 to suppress motility of ovarian carcinoma cells^12^, knockdown of GM2/GD2-synthase reduced tumor cell migration, motility and invasiveness^13^, suggesting complex and differential roles of gangliosides in tumorigenesis.

Despite these pathologically significant roles of select tumor derived gangliosides, the precise biological role of certain gangliosides like GM2 and GD1a in modulating tumor cell behavior still remains unclear. Since, gangliosides like GM2 and GD1a are found to be aberrantly over-expressed and secreted in a variety of tumors, the immediate need is to study the outcome of such an over-expression and define the role of these gangliosides in tumorigenesis. Previously, ganglioside knockout mice were generated to study the role of gangliosides in neuronal development, immune effector cell maturation and development^14^,^15^,^16^. However, knockout mice model may not be ideal to study the role(s) of gangliosides in tumorigenesis, since these models constitute a systemic knockout of total or select gangliosides, which is usually not reflected in the case of physiological tumorigenesis. Ganglioside knockout mouse embryonic fibroblasts (MEFs) were reported in few studies to unravel the role of gangliosides in carcinogenesis^2^,^17^. However, studies using ganglioside over-expressing cancer cells and its ganglioside knockout counterparts will actually enable us to clearly understand how over-expression of select gangliosides alter tumor cell behavior. To address this issue, we generated specific ganglioside knockout mouse cancer cell line to study its role in neoplastic transformation. Further, generation of a stable GM2-synthase knockout murine cancer cell line provides a scope of assessing the role of these gangliosides *in vivo*. Precise and targeted genome engineering approach using transcription activator-like effector nucleases (TALENs) technology was applied to specifically knockout murine GM2/GD2-synthase gene. TALENs are tandem array of TALE repeats fused with Fok1 nuclease domain, which can recognize any given DNA with high specificity over Zinc Finger Nucleases (ZFNs) and CRISPR/Cas9 system^18^. TALEN construction is also easy and economical as compared to ZFN^19^. Also, TALEN mediated off-target cleavage and nuclease associated cytotoxicity is extremely low^19^,^20^. TALEN induced double strand break was reported in various organisms including yeast, nematodes, annelids, arthropods, fish, amphibians, mouse, rat, monkeys, cultured mammalian somatic cells and stem cells^21^,^22^,^23^.

In an attempt to study GM2 mediated tumorigenesis, we report here designing of GM2-synthase specific TALEN to efficiently knockout GM2 and complex gangliosides. Constructed TALENs induced mutation at GM2-synthase TALEN target site with a consequent total depletion of GM2 in Renca-v cells, which have been shown to over-express GM2 (supplementary figure S2a) over a normal mouse fibroblast cell line (12)1/CA^24^. GM2 knockout clones (Renca-v^GM2 synthase−/−^) showed no change in cellular proliferation and clonogenicity but exhibited significantly reduced anchorage-independent growth (AIG) and higher adhesion to extracellular matrix protein by causing a loss in anoikis resistance confirming a role of GM2 and downstream complex gangliosides in neoplastic transformation and metastatic ability of the cells. Moreover, TALEN mediated specific ganglioside knockout caused a moderate yet significant reduction in tumor volume in syngeneic Balb/c mice. Further, this syngeneic model will also help in understanding the function of tumor shed/secreted gangliosides in modulation of the tumor microenvironment as well as immune effector cell dysfunction *in vivo.*

## Results

### Construction, over-expression and functional validation of TALEN targeted against GM2-synthase in mouse cell lines

**Figure 1:**
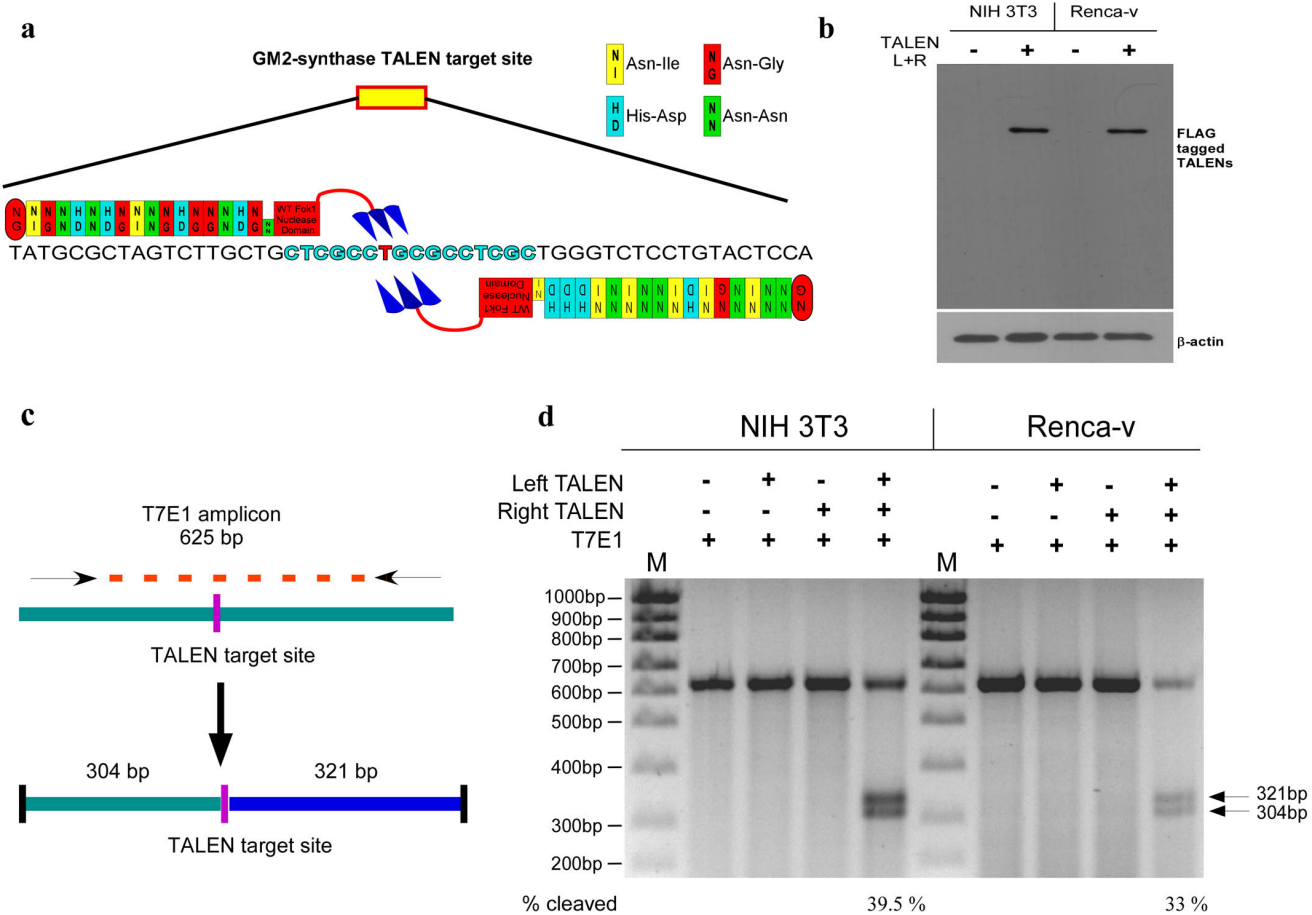
Design, construction and validation of mouse GM2-synthase specific TALEN pair. *Fig. 1a* shows exemplified GM2-synthase TALEN target region with target DNA sequence. DNA sequence with black letters indicates TALEN target sequence against which TALEN pair has been designed; blue letters represent spacer DNA sequences and red letter specify the target base position of Fok1 dimerization. TALEN modules are represented as yellow, red, green or blue boxes according to their base recognition specificity of A,T,G or C respectively. Large red box with overhanging 3 arrows indicates wild type Fok1 nuclease domain. *Fig. 1b* Western immunoblotting was done to detect over-expression of FLAG tagged TALEN pair in mouse NIH 3T3 and Renca-v cells, using anti-FLAG antibody. β-actin was used as loading control (image of β-actin blot was cropped and original blot shown in supplementary information). *Fig. 1c* shows a schematic representation describing PCR amplification of TALEN target region and resulting two fragments arising from digestion of DNA-heteroduplexes by T7E1 enzyme. T7E1 assay was conducted to detect gene editing activity of GM2-synthase TALEN pair. *Fig. 1d* shows PCR amplified genomic DNA from TALEN pair transfected (left + right), single TALEN transfected (left or right), or non-transfected NIH 3T3 or Renca-v cells and subjected to digestion with T7E1 enzyme. T7E1 digested products as well as size of the DNA fragments were indicated by arrows (original gel image shown in supplementary information). Gels have been run under same experimental conditions.

Since the aim of the study was to establish a stable and permanent GM2-synthase knockout mouse cell line in order to define the role of complex gangliosides in mediating tumorigenesis, specific TALEN pairs were designed and constructed using restriction enzyme and ligation (REAL) method as described before^25^. A genomic region within the GM2-synthase locus of mouse chromosome 10, present in the coding sequence (CDS) of all four transcript variants of mouse GM2-synthase mRNA was selected and targeted as shown in Fig. 1a. Here, a region conserved in exon 2 of transcript variant 1 and 3 and exon 1 of transcript variant 2 and 4 were specifically targeted as shown in supplementary figure S1a. Left and right TALENs were constructed by serial digestion and ligation of the TALE repeats, as shown in supplementary figure S1b and S1c and described in methodology^25^. To assess the function of constructed TALEN pairs, TALEN expressing plasmids were co-transfected in equal amounts in two mouse cell lines NIH3T3 and Renca-v. Western immunoblotting was performed 48 hr's post transfection to check TALEN expression. Fig. 1b clearly indicates that TALEN pair was over-expressed in both NIH3T3 and Renca-v cells transfected with TALEN pairs as compared to non-transfected cells. In order to find out whether TALEN over-expression actually targeted GM2-synthase, the functionality of the TALEN pairs was checked for their ability to create insertion or deletion mutations (indels) at the GM2-synthase target region by T7E1 assay^26^. For this, the genomic region covering the TALEN target region was PCR amplified as shown in Fig. 1c followed by digestion with T7E1 endonuclease. Fig. 1d shows that our constructed TALEN pair was functional only when both the left and right TALENs were co-transfected as indicated by two cleavage bands in both NIH3T3 (cleavage 39.5%) and Renca-v (cleavage 33%) cells. However, transfection of either one of these TALENs into the cell lines did not show any cleavage activity, indicating that both left and right TALENs were required for its activity.

### Analysing potential off-target effect of TALEN targeted against mouse GM2-synthase

**Figure 2:**
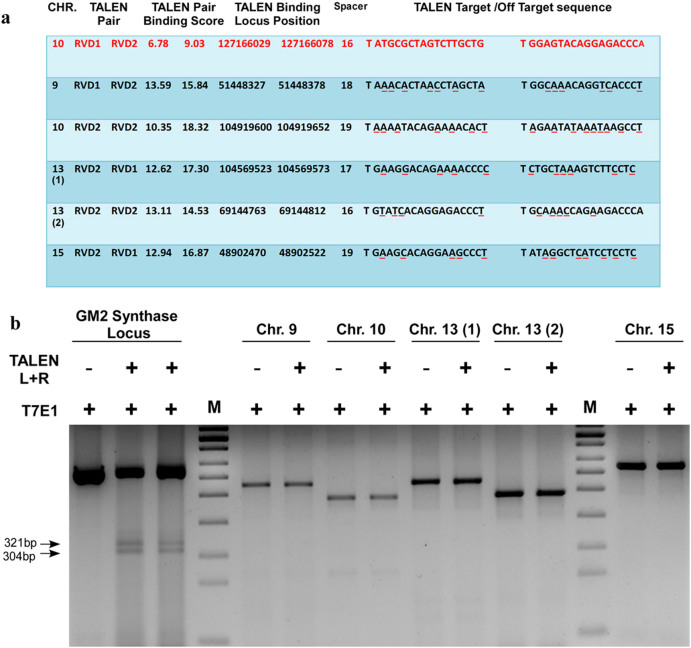
Analysis of off-target effect mediated by GM2-synthase TALEN pair. *Fig. 2a* shows tabulated form of five off-target DNA sequences from different chromosomes obtained from searching the GM2-synthase TALEN pairs against the mouse genome using the “Paired Target Finder” tool of online “TAL Effector Nucleotide Targeter 2.0”. A lower TALEN pair binding score indicated higher binding affinity between RVDs and target DNA sequences. The upper first row in red letters shows the GM2-synthase target region in chromosome 10 with lower binding score. In other rows, letters with red underline denotes mismatches with original TALEN binding sequence. *Fig. 2b* Five off-target regions were PCR amplified from mouse genomic DNA and subjected to digestion with T7E1 enzyme. Detectable gene editing activities by GM2-synthase TALEN pair was not observed in these five potential off-target genomic loci in Renca-v cell line as indicated by digestion with T7E1 enzyme, indicating that the constructed GM2-synthase TALEN pairs were strictly sequence specific and did not have any significant off-target effects. Image of agarose gel was cropped and original gel image shown in supplementary information.

Since, wild-type Fok1 nuclease domain was used in the TALEN expression vector during the construction of TALEN pairs, constructed TALEN pairs might bind non-specifically to any genomic region other than the target as dimerized wild-type Fok1 has the ability to induce cleavage anywhere in the genome. Hence, we searched for potential off-target effect of GM2-synthase TALEN pairs throughout the mouse genome using the “Paired Target Finder” tool of online “TAL Effector Nucleotide Targeter 2.0” as described earlier^26^. No potential off-target regions with complete sequence match and equal TALEN pair binding scores were found. However, result from this tool identified huge number of off-target regions in the mouse genome with different TALEN binding scores from which five off-target regions were selected from different chromosomes with low TALEN binding scores in different combination of RVD (Repeat Variable Di-residues) monomer. Fig. 2a represents the off-target binding sequence with TALEN pair binding scores. To check the off-target effect of GM2-synthase TALEN pairs, a genomic region spanning the off-target region along with GM2-synthase target region from either wild-type or TALEN transfected Renca-v cell line was PCR amplified and then subjected to digestion with T7E1 enzyme. Fig. 2b clearly indicates that GM2-synthase TALEN pairs showed no detectable off-target effect as measured by T7E1 assay in at least five different sites in different chromosomes as compared to GM2/GD2-synthase locus where TALEN pairs specifically induced gene editing activity indicated by two cleavage bands. Since, the five off-target sequences were chosen on the basis of lowest binding score (highest binding affinity) amongst the predicted off-target sequences, negative T7E1 data from these five off-target sequences confirm that our designed TALEN pairs against GM2-synthase induced gene editing activity in a strictly sequence-specific manner.

### Generation of stable GM2-synthase knockout Renca-v cell line

**Figure 3:**
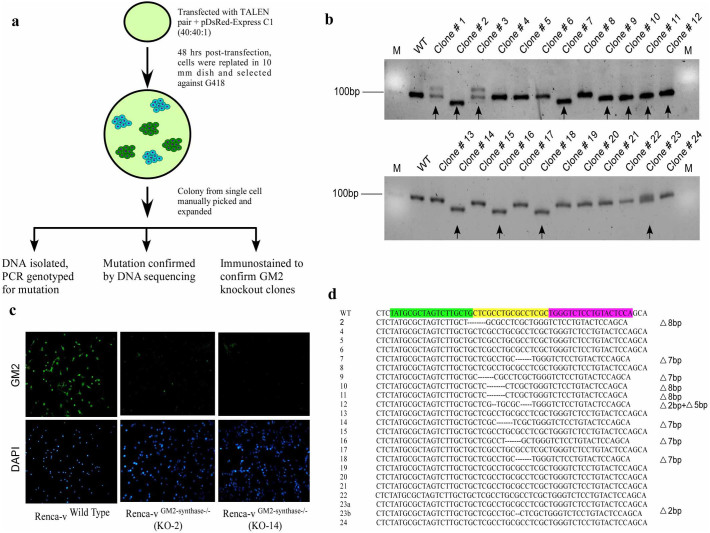
Generation and characterization of GM2-synthase knockout Renca-v cell line. *Fig. 3a* shows a schematic representation of the strategy used for generation of TALEN mediated GM2-synthase knockout Renca-v cell line. Renca-v cells were transfected with TALEN pairs and pDsRed-Express C1 empty vector (containing a neomycin/G418 resistance cassette) at 40:40:1 and selected against G418. Each single colony was then manually picked, expanded and genomic DNA was isolated from each expanded colonies. PCR genotyping was performed to detect TALEN mediated mutations, while indel mutations were confirmed by sequencing. Complete GM2 knockout clones were selected and expanded on the basis of GM2 expression by immuno-staining. *Fig. 3b* represents PCR genotyping from selected 24 clones using high resolution metaphor agarose. Black arrows indicate mono-allelic or bi-allelic mutants. Clone 1, 3, 23 represents mono-allelic mutation. Clone 2,7, 9–12, 14,16 and 18 shows bi-allelic mutation (metaphor agarose gel images was cropped to show DNA band encompassing PCR amplified TALEN target region and original gel images shown in supplementary information. Both the gels were run on the same experiment following same experimental conditions). Stable GM2-synthase knockouts were finally selected and expanded on the basis of ganglioside GM2 expression by immuno-staining of GM2 in Renca-v wild type and knockout clones, as shown in *Fig. 3c*. No expression of GM2 was observed in the two knockouts (KO-2 and KO-14) clones as compared to wild type clones. *Fig. 3d* shows sequence analysis of 22 clones which identified different indel mutations in the TALEN target region. Deletions are indicated as black dashes.

Although, several strategies exist to knockdown expression of a target gene at the cellular level, particularly siRNA transfection, however, these strategies posed a serious limitation as knockdown was mostly transient. In fact, data from our laboratory suggests a successful yet transient siRNA mediated knockdown of GM2 expression in Renca-v cells as shown in supplementary figure S2b, which clearly demonstrated reversal of GM2 expression at 72 hr's. Hence, the rationale for generation of complete GM2-synthase knockout Renca-v cells using GM2-synthase specific TALENs. Renca-v cells were chosen based on the over-expression of GM2 by these cells compared to normal mouse cell line (12)1/CA (fibroblasts) which shows negligible expression of GM2 as shown in supplementary figure S2a. Renca-v cells were co-transfected with TALEN pairs and pDsRed-Express C1 empty vector (to select the cells on the basis of neomycin/G418 selection) at a ratio of 40:40:1^27^ and clonally selected as described in materials and methods (Fig. 3a). Genomic DNA was isolated from each of these clonally selected and expanded 24 clones and PCR genotyping was performed to check TALEN induced mutation at the GM2-synthase target region. PCR genotyping primers were designed to amplify a small portion (101 bp) of GM2-synthase locus flanking the TALEN target region. Fig. 3b depicts PCR genotyping data from all 24 isolated clones, clearly indicating shifting of GM2-synthase target amplicon in several clones as compared to wild type amplicon, thereby confirming TALEN mediated indel mutation at the GM2-synthase target region. Further, as we aimed to establish complete GM2 knockout Renca-v cell line, immuno-fluorescent staining followed by microscopy was performed to check GM2 expression in all 24 clones. Results indicate that four clones among the 24 had complete depletion of GM2, two of which (KO-2 and KO-14) were shown in Fig. 3c. In order to identify the inserted or deleted bases from the TALEN target region, 425 bp genomic region flanking GM2-synthase TALEN target region was PCR amplified. PCR product was then gel eluted and cloned into TA cloning vector pTZ57R/T and directly sequenced using T7 forward and M13/pUC sequencing primers. Sequencing of 22 clones showed that indel mutations occurred in 10 clones, with deletions ranging from 2–8 bases as shown in Fig. 3d.

### NHEJ mediated integration of a neomycin cassette at TALEN targeted GM2-synthase locus

**Figure 4:**
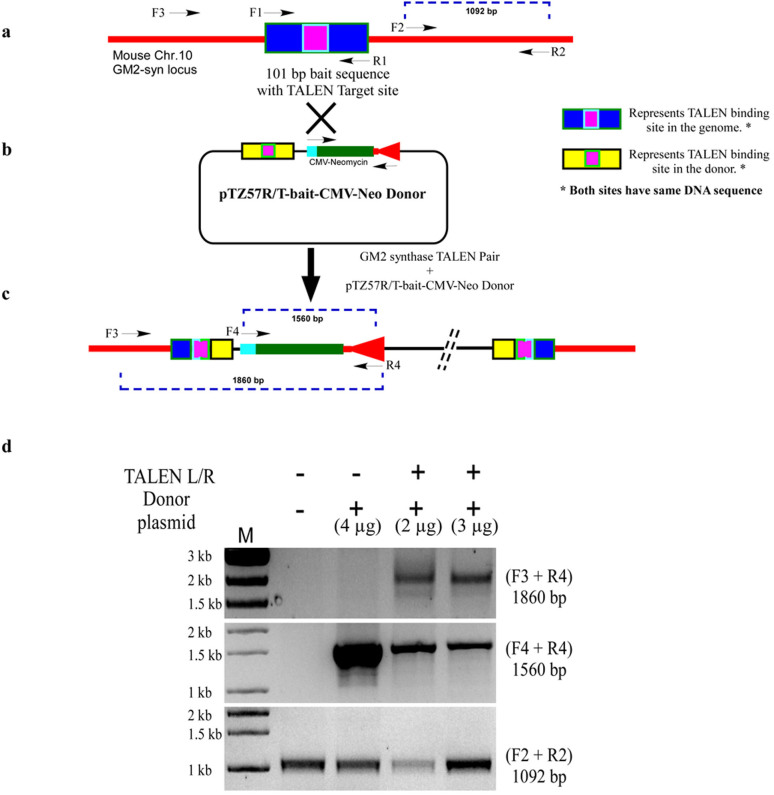
TALEN mediated targeted integration of CMV-Neomycin cassette into the GM2- synthase locus. *Fig. 4a.* shows a schematic representation of murine GM2-synthase locus. Blue box indicates the 101 bp genomic region which was amplified to design the donor vector. Pink box with green border (inside the blue box) indicates TALEN target region. *Fig. 4b.* A schematic of donor plasmid pTZ57R/T-bait-CMV-Neo. CMV-driven neomycin cassette was first cloned into Apa1 site of self-circularized TA cloning vector pTZ57R/T. Then the 101 bp bait sequence was cloned into the C-terminal of neomycin cassette. Yellow box indicates the bait sequence in the donor vector, pink box (inside the yellow box) indicates TALEN binding site. *Fig. 4c. A* schematic showing NHEJ-mediated integration between TALEN targeted GM2-synthase locus and the donor vector. Junction PCR using primer set (F3 + R4) was used for assessing targeted integration. Blue dotted line indicates the size of PCR products. *Fig 4d.* A representative agarose gel analysis of PCR genotyping demonstrating targeted integration of neomycin cassette into GM2-synthase locus (all agarose gels were cropped and original images shown in supplementary information. All individual gels were run under same experimental conditions). Genotyping PCR was performed to assess the integration event from Renca-v cells transfected with different amount of donor plasmid with fixed amount of TALEN pair, only donor plasmid or non-transfected cells. Primer pair (F3 + R4) was used to perform the junction PCR, primer pair (F4 + F4) was used to amplify the neomycin cassette and primer pair (F2 + R2) was used to amplify a part of GM2-synthase beyond TALEN target region.

Since, non-homologous end joining (NHEJ) is a predominant DNA repair mechanism in cancer cells, TALENs, CRISPR and ZFNs may act as valuable tools to introduce a desired gene cassette to the specific genomic locus. Hence, we opted for homology-independent targeted integration of a CMV-neomycin cassette into the GM2-synthase TALEN target site^28^. To achieve NHEJ mediated integration of a genomic cassette, a donor vector was constructed in which 101 bp TALEN target region or bait sequence (Fig. 4a) was cloned followed by CMV-driven neomycin cassette in a TA cloning vector, lacking any homology to the GM2-synthase locus as represented in Fig. 4b. When donor plasmid was co-transfected with TALEN pair, concurrent cleavage of TALEN target region at genomic loci and donor plasmid at TALEN target region occurred, resulting in integration of the donor plasmid at the GM2-synthase target site as shown in Fig. 4c. 72 hr's post-transfection, integration of donor plasmid at the GM2-synthase target locus was assayed by junction PCR, applying one primer from genomic loci beyond TALEN target region (Primer F3 in Fig. 4c) and one primer from targeted donor vector (Primer R4 in Fig. 4c). Successful and targeted integration of donor vector in right orientation in the two experimental conditions were achieved as shown in Fig. 4d, where two different concentrations of donor plasmids were used as compared to only donor vector transfected and non-transfected Renca-v cells. This experiment helped us in not only achieving disruption of GM2-synthase target, but simultaneously mediate integration of a neomycin cassette at the GM2-synthase genomic locus. This will enable us to drive TALEN mediated targeted insertion of a fluorescent cassette (GFP/RFP/Luciferase) at the GM2-synthase locus to eventually generate fluorescently tagged GM2-synthase disrupted cells that could be used to study the role of this gene in metastasis *in vivo*.

### GM2-synthase knockout Renca-v cells exhibited a significant decrease in anchorage-independent growth and reduction in tumor volume

**Figure 5:**
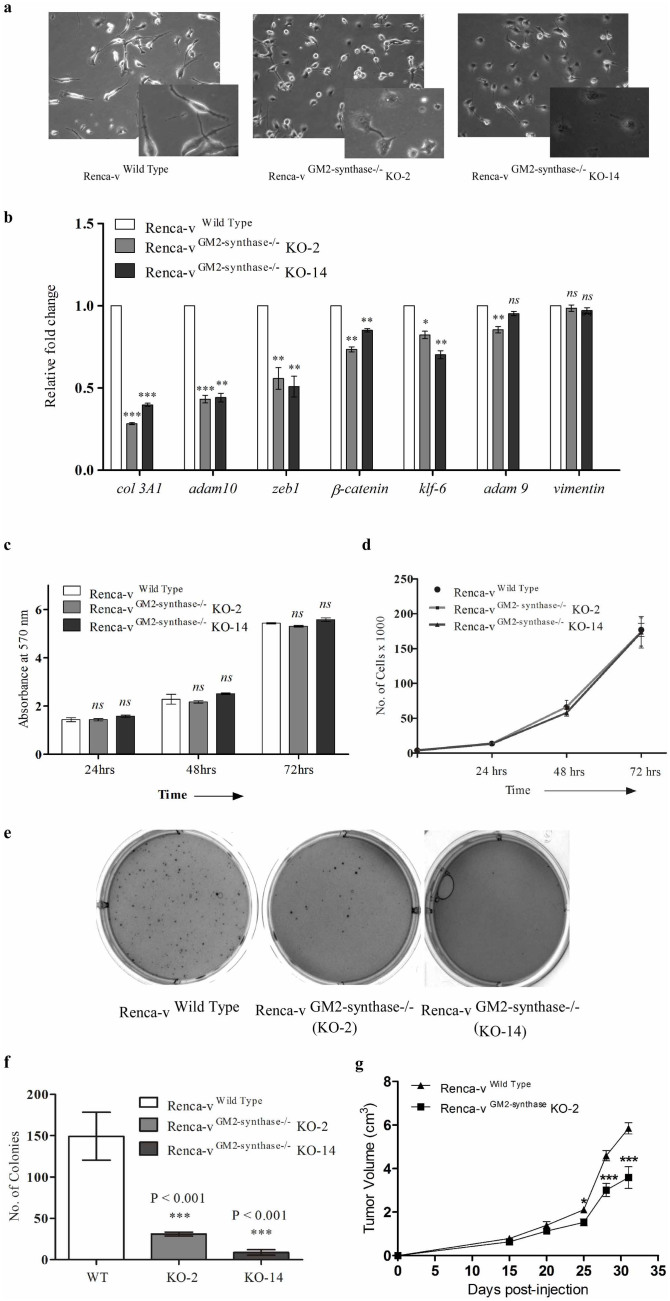
Permanent and stable disruption of GM2-synthase resulted in significant reduction in AIG and tumor growth without affecting cellular proliferation. *Fig. 5a*. Phase contrast microscopic image showing morphology of Renca-v wild type and two GM2-synthase knockout clones. Mesenchymal to epithelial phenotypic transition in response to GM2-synthase knockout was confirmed by expression analysis of epithelial and mesenchymal markers in the knockout clones versus the wild type Renca-v cells by real time PCR (*Fig. 5b*). Real-time PCR was performed in triplicate and expressed as the fold differences of target gene expression relative to the wild type. *In vitro* cellular proliferation of Renca-v wild type and two GM2-synthase knockout clones were measured either by using MTT assay *(Fig. 5c)* or by counting cells at 24 hr's, 48 hr's and 72 hr's using hemocytometer *(Fig. 5d)*. Results are mean ± SEM. p-values: WT vs KO-2 - 0.9872 (24 hr's), 05162 (48 hr's), 0.0737 (72 hr's) and WT vs KO-14 - 0.1299 (24 hr's), 0.2531 (48 hr's), 0.3836 (72 hr's). Anchorage independent growth (AIG) of cells was measured by soft agar colony formation assay *(Fig. 5e *&* 5f)*. Renca-v and the two GM2-synthase knockout clones were grown on soft agar plates for 15 days before colonies were stained with crystal violet and imaged. Images from one representative experiment were shown in *Fig. 5e.* Graphical representation of the number of colonies counted from wild type and two GM2-synthase KO clones were shown in *Fig. 5f*. Total number of colonies were counted from four wells (duplicate wells from each independent experiment). Results are mean ± SEM. *** P < 0.001 vs WT. Renca-v WT and KO-2 cells were used to develop tumors in syngeneic Balb/c mice and tumor formation was monitored by measuring tumor volume with time as shown in *Fig. 5g*. Tumor volume was measured at regular intervals till day 31st post injection of tumor cells. Results are mean ± SEM of 2 independent experiments with at least 8 mice per group at every time point. *P < 0.05 vs Renca-v WT (at day 25), and ***P < 0.001 vs Renca-v WT (at day 28 and 31).

Permanent and stable GM2-synthase knockout clones (Renca-v^GM2-synthase−/−^) were used to define the potential role of GM2 in tumorigenesis. To determine the cellular phenotype modulated by knockout of GM2-synthase, we compared cellular morphology and proliferation rate of wild type versus the two Renca-v knockout clones. Whereas Renca-v wild type cells showed elongated and spindle shaped morphology with higher number of cellular protrusions, most of the cells from the two knockout clones showed rounded and more epithelial phenotype with very few number of cellular projections as shown in Fig. 5a. This was further confirmed by expression analysis of key genes involved in epithelial-mesenchymal transition^29^,^30^,^31^,^32^,^33^ by RT-PCR (Fig. 5b). Data clearly indicates a considerable as well as significant reduction in the mRNA levels of key mesenchymal marker genes, *col3A1, adam10* and *zeb1*, while *β-catenin* and *klf-6* showed a moderate yet significant downregulation in the two GM2-synthase KO clones versus the wild type Renca-v cells, thereby confirming that GM2-synthase knockout drive the cells to become more epithelial. Although, *adam9* and *vimentin* are mesenchymal markers^29^,^31^, however, they did not show any considerable change in their expression in the GM2-synthase knockout clones as shown in Fig. 5b as compared to the wild type. In order to find out whether Renca-v^GM2-synthase−/−^ exhibited altered cellular proliferation, time dependent (24, 48 and 72 hr's) proliferation was monitored by MTT assay^34^ as shown in Fig. 5c and cell counting assay (Fig. 5d) in the two knockout clones versus the wild-type Renca-v cells. Data clearly shows that disruption of the GM2-synthase gene did not affect cellular proliferation significantly in the two clonally selected Renca-v^GM2-synthase−/−^ cell lines as compared to wild-type Renca-v cells. Since most cancer cells inherit the property of anchorage independence which allow them to grow without the need of any anchorage^35^, we wanted to know whether GM2-synthase knockout from cancer cells would affect their anchorage independence. Hence, soft agar anchorage-independent growth assay was performed and anchorage independence was assessed both by the number of foci formed and also the size of individual colonies. Results showed that the two GM2 knockout Renca-v^GM2-synthase−/−^ cells exhibited a dramatic reduction (Fig. 5e and Fig. 5f) in the number of colonies, indicating a significant decrease in AIG, as well as smaller colonies (supplementary Fig. S3) as compared to wild-type Renca-v cell line, suggesting a role of GM2 in neoplastic transformation. We further assessed the effect of GM2-synthase knockout on primary tumor growth in a syngeneic mouse model using Balb/c mice as described earlier^36^,^37^. While the Renca-v wild type cells formed solid tumors that increased with time, Renca-v^GM2-synthase−/−^ (KO-2) cells caused a moderate (~ 34% on day 28th and ~ 38% reduction on day 31st) yet significant reduction in tumor volume, as shown in Fig. 5g.

### GM2-synthase knockout Renca-v cells showed higher adhesion to fibronectin and less anoikis resistance

**Figure 6:**
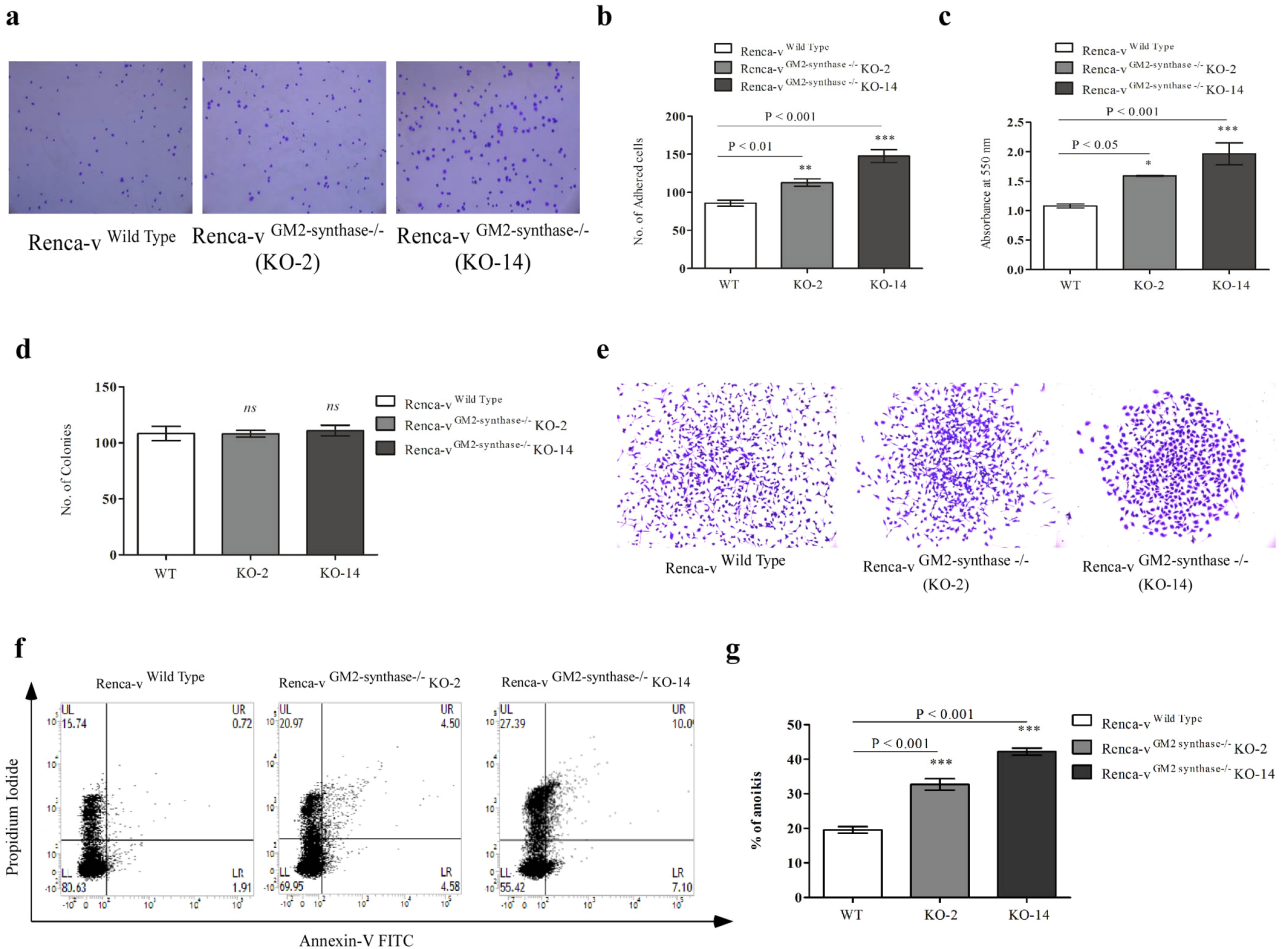
GM2-synthase knockout results in increased cellular adhesion and loss of anoikis resistance of Renca-v cells. *Fig. 6a.* represents the microscopic image showing adhered cells to fibronectin coated plates. In brief, equal number of cells were plated over fibronectin coated plates and incubated for 15 mins, stained with crystal violet and photographed. *Fig. 6b.* shows graphical representation of the number of cells adhered (9 microscopic fields) to fibronectin, counted from wild type and two GM2-synthase KO clones. *Fig. 6c.* graphical representation of adhered cell as determined by O.D value at 550 nm, following addition of 2% SDS to crystal violet stained cells and incubation for 30 mins. *Fig. 6d.* shows graphical representation of number of colonies in clonogenicity assay. Experiments were performed three times in triplicate. *Fig. 6e.* represents the clonal morphology of Renca-v wild type and two GM2-synthase knockout clones. *Fig. 6f.* Representative flow cytometry image showing % anoikis in Renca-v wild type vs two GM2-synthase knockout clones at 24 hr's time point. The experiments were performed three times in triplicate. *Fig. 6g.* Graphical representation of anoikis induced cells in Renca-v wild type and two GM2-synthase knockout clones. Results are mean ± SEM. *P < 0.05 vs WT, **P < 0.01 vs WT, *** P < 0.001 vs WT.

Since, loss of cellular adherence had been associated with increased metastatic ability of tumor cells^38^, we wanted to check whether knockout of GM2-synthase in these two clones would affect cellular adherence. To test this hypothesis, we performed replating cell adhesion experiments. Disruption of the GM2-synthase gene increased cell adhesion to fibronectin significantly in the two clonally selected Renca-v ^GM2-synthase−/−^ cell lines as compared to wild-type Renca-v cells as shown in Fig. 6a, 6b & 6c, suggesting that GM2 and complex gangliosides helped the cells to lose adherence which might contribute towards increased metastatic ability. Further, when cells were plated in very low density in standard two-dimensional cell culture dishes and cultured for 5–7 days, no significant difference was observed in the clonogenicity (colony formation ability) of Renca-v (wt), KO-2 and KO-14 (Fig. 6d & supplementary Fig. S4), but interestingly individual clonal shape and morphology were phenotypically distinctly different. Fig. 6e shows that while Renca-v wild type clones were dispersed and have no defined structure, KO-2 and KO-14 clones were compact and have defined structure displaying a more epithelial phenotype.

Enhanced anchorage independent growth of cancer cells reflect their resistance to anoikis^39^. It was possible that upregulated GM2 in cancer cells might provide protection for the cells from undergoing anoikis, leading to event of molecular signalling pathways which help in gaining anchorage independence. To test this hypothesis, we compared the detachment induced cell death of Renca-v (WT) cells and two Renca-v^GM2-synthase−/− ^knockout clones. For this, cells were grown onto low attachment plates (to inhibit cell-matrix adhesion) in the presence of 1.25% methyl cellulose (to inhibit cell-cell adhesion) and then FACS based assay was performed to determine cell death using an apoptosis assay kit. Following 24 hr's of suspension culture, both Renca-v clones, KO-2 (>35% death) and KO-14 (>40% death) showed significantly increased cell death compared to the wild type which shows ~ 20% cell death (Fig. 6f & Fig. 6g) as evidenced by increased propidium iodide staining by the KO clones compared to the wild type cells, indicating a significant loss in anoikis resistance in the two knockout clones. Together, these data strongly support the role of GM2 in acquiring anchorage independence, cancer cell adhesion, and anoikis resistance.

## Discussion

In order to understand the functional relevance of a particular gene in a disease context, genetically modified animals and cells are essentially useful in basic and advanced biomedical research. siRNA mediated silencing of a target gene or simply plasmid-mediated gene cloning and transfection were previously attempted for genetic manipulation of cells. However these techniques were successful in producing only a transient effect. Permanent integration/knockout of a gene has been achieved by virus-mediated (retrovirus, adenovirus or baculovirus) gene transduction^40^,^41^,^42^ or cre-lox^43^ mediated transgenesis. Most of these strategies were laborious, and frequency of targeted integration was extremely low. Recently, ZFNs, TALENs and CRISPR/Cas mediated genome editing methods have emerged as a major tool for targeted genome editing and are now widely used for their ease of design, construction and significantly higher editing frequency.

We anticipated the rapid advancement of TALEN mediated genome engineering technology and applied this technique to generate a permanent and stable GM2-synthase knockout cell line in order to investigate the functional role of this gene in tumorigenesis. Although several high throughput methods for automated TALEN construction have been reported earlier^44^, in the present study we applied the REAL method (developed by Joung lab) to assemble the TALEN pairs. REAL method for TALEN construction is user-friendly, cost-efficient, non-PCR based simple restriction enzyme and ligation method which have already been tested to perform non-homologous end joining (NHEJ) with high frequencies in a variety of organisms and cell types^44^. Here, we demonstrated the feasibility of genome engineering tool, TALEN, to knockout the murine GM2/GD2-synthase gene from a GM2-overexpressing variant of a mouse kidney cancer cell line (Renca-v) to study the function of this gene in cancer. TALEN pairs were strategically designed against a target region present in the coding sequence of all four transcript variants of mouse GM2-synthase as indicated by the yellow box in Fig. 1a and supplementary figure S1a. Further, the TALEN target region in all four transcript variants were strategically chosen so that it falls immediately downstream of translation start site of GM2-synthase to ensure maximal probability of a disrupted and non-functional protein. Using the above strategies, we successfully designed and assembled the mouse GM2/GD2-synthase specific TALEN pairs using REAL method which showed gene editing activity in two different murine cell lines, as demonstrated by T7E1 assay (Fig. 1c & 1d). The specificity of the TALEN pairs was tested, since a possibility exists for “off-target” cleavage of the genome owing to the non-specific activity of Fok1 endonuclease flanking each TALE. Predicted off-target binding sites of the constructed TALEN pairs were obtained from the “Paired Target Finder” online tool as described before and shown in Fig. 2a. This was again experimentally verified by a T7E1 assay with five different off-target sequences located in mouse chromosome 9, 10, 13 and 15 as depicted in Fig. 2b. Overall, this was indicative of the fact that the constructed TALEN pairs were efficient in disrupting the GM2-synthase gene at the target site in a strictly sequence specific manner. Clonal selection followed by PCR genotyping and sequencing confirmed both mono- and bi-allelic mutations in Renca-v cell lines, as observed by single and double bands in Fig. 3b with an indel mutation frequency at GM2/GD2-synthase locus of around 45%. Using a modified clonal selection strategy (Fig. 3a) to isolate potential TALEN targeted GM2-synthase knockout clones, enabled us to obtain a mutation frequency which was significantly higher than the TALEN induced mutation rates in human cell lines reported previously^44^. We further anticipated that generation of a stable GM2-synthase knockout cell line that would also express a selection marker (like GFP/RFP/luciferase/neomycin) would not only help in efficient and faster selection of knockout clones but would also immensely help in studying the role of complex gangliosides in metastasis *in vivo* in future experiments. For this, a gene cassette needs to be integrated at the specific GM2-synthase genomic locus targeted by TALEN. Emerging reports have shown that ZFN, TALEN and CRISPR/Cas9 mediated targeted integration of a desired gene cassette in cultured mammalian cells^28^,^45^ and zebrafish^46^ via homology independent break repair. This process of targeted integration does not rely on homology directed repair, rather depends on NHEJ for integration of foreign DNA^28^. As NHEJ is a predominant DNA repair mechanism in cancer cells, we attempted integration of a CMV-neomycin cassette into the GM2-synthase TALEN target site. Data shown in Fig. 4d clearly depicts that our constructed TALENs mediated integration of a neomycin cassette at the targeted GM2-synthase genomic locus.

siRNA mediated silencing of ganglioside synthases, exogenous ganglioside treatment or pharmacological inhibitor-based approach to inhibit total ganglioside synthesis were previously used to demonstrate the role of gangliosides in tumorigenesis. However, the major limitation of these studies lies in their ability to cause only transient inhibitions of ganglioside biosynthesis. Our study using mouse GM2/GD2-synthase siRNA transfection in Renca-v cell line showed transient knockdown of GM2-synthase mRNA with a parallel time-dependent decrease in ganglioside GM2 expression, however, at 72 hr's GM2 expression reverted back (supplementary Fig. S2b). Pharmacologic inhibitors of ganglioside biosynthetic pathway have also been well studied. Although PDMP, a glucosylceramide synthase inhibitor^47^ was found to inhibit tumor growth and metastasis in Lewis lung carcinoma model^48^, PDMP treatment in cultured cells showed proliferation inhibition followed by accumulating ceramide induced apoptosis^49^. PPPP, a derivative of PDMP, shown to be a more specific inhibitor of glucosylceramide synthase neither affected cell proliferation *in vitro* or induced apoptosis mediated by ceramide accumulation, however withdrawing the PPPP from the culture medium caused complete reversal of ganglioside biosynthesis after 3 days^50^. Hence, these strategies to manipulate the ganglioside biosynthesis had limitations for *in vivo* study. Furthermore, using pan ganglioside synthesis inhibitors makes it extremely difficult to dissect the differential roles, each ganglioside might play in tumor growth, proliferation and metastasis. In order to address these issues we adopted a specific and targeted knockout approach to disrupt ganglioside synthase genes and subsequently specific gangliosides permanently, to obtain tumor cells lacking expression of a specific series of gangliosides. Functionally, TALEN mediated knockout of GM2-synthase and consequent GM2 expression, showed morphological differences (Fig. 5a) with the KO clones showing a more epithelial phenotype compared to the wild type Renca-v cells. This mesenchymal to a more epithelial phenotype upon GM2-synthase knockout was further confirmed by expression analysis of key markers involved in EMT as shown in Fig. 5b. Further, the GM2-synthase KO clones demonstrated significant reduction in anchorage independent growth (Fig. 5e & 5f) as well as colony dimension (supplementary Fig. S3) as compared to wild-type Renca-v cells, thereby indicating a critical role of GM2 in oncogenic transformation. Moreover, the GM2-synthase knockout clone KO-2 demonstrated moderate yet significant reduction in tumor volume with time in a syngeneic mouse tumor model, as shown in Fig. 5g thereby confirming the role of GM2 and downstream complex gangliosides in tumorigenesis. However, the fact that both wild-type and GM2-synthase knockout cells showed no significant alterations in cell proliferation or cell number as shown in Fig. 5c & 5d, might suggest the use of a metastatic tumor model rather than a localized model^51^ to investigate the *in vivo* role of GM2-synthase or complex gangliosides (including GM2) in future studies. For cancer cells to become anchorage independent, they need to lose adherence, detach and grow independent of anchorage. Hence, our data in Fig. 6a, 6b & 6c showing increased cellular adherence in GM2-synthase knockout clones KO-2 and KO-14 over the wild type cells suggests a possible role of GM2 in promoting loss of adherence in tumor cells, which might in turn contribute towards the metastatic ability of the tumor cells. Cancer cells grow anchorage independently due to their inherited ability to resist anoikis, a form of cellular death induced by detachment^52^. Our data in Fig. 6f & 6g demonstrating increased susceptibility of the KO clones to anoikis provided the first evidence suggesting the ability of GM2 and complex gangliosides in promoting anoikis resistance and hence allowing the tumor cells to grow anchorage independently.

Overall, we have developed a TALEN based targeted gene editing strategy to knockout ganglioside synthase gene in murine GM2 over-expressing cancer cell line. These GM2/GD2- synthase knockout cells showed complete loss of GM2 expression. TALEN-mediated knockout of GM2/GD2-synthase inhibited anchorage-independence by increasing cellular adherence and promoting anoikis, and caused reduction in tumor growth in mice. Hence, we identified GM2/GD2-synthase (over-expressed in many cancer types), as a critical enzyme to modulate cancer cell behavior by promoting their ability to survive anchorage-independently by mediating loss of adherence and promoting anoikis resistance in tumors. From these findings, we could speculate that high expression of GM2 promotes anchorage-independent growth of tumors, which is a pre-requisite for neoplasticity and TALEN mediated knockout inhibited this process thereby providing an opportunity for therapeutic intervention for inhibiting the metastatic potential of cancer cells. So, the results presented in this study not only reveal the functional roles complex gangliosides, including GM2 play in mediating tumorigenesis and highlights ganglioside GM2 as a target for developing anti-cancer therapies, but also sheds light on a new class of genome editing tool, TALEN with tremendous potential for gene therapy in cancer.

## Methods

### Reagents and Chemicals

TALEN kit (ID-1000000017) was purchased from Addgene (Cambridge, MA, USA). Restriction enzymes, quick ligation kit, Q5 DNA polymerase, T7E1 enzymes were purchased from NEB (MA, USA). DNA ladders and dNTPs were purchased from NEB as well as from Biobharati Life Sciences, Kolkata, India. Ex-Taq was purchased from Takara (Japan). High resolution metaphor agarose was purchased from Lonza (Rockland, USA). pTZ57R/T was obtained from Thermo Scientific (EU, Lithuania). All antibiotics, cell culture reagents and MTT were purchased from Hi-Media (Mumbai, India) and FBS, opti-MEM, lipofectamine from Invitrogen (CA, USA). Low attachment plates (LAPs) were purchased from Corning (NY, USA). Methyl cellulose, mouse monoclonal anti-FLAG Ab and HRP-goat anti-rabbit IgG (2°Ab) was obtained from Sigma-Aldrich (St. Louis, USA). Rabbit polyclonal β-actin Ab was purchased from Imgenex, Bhubaneswar, India. Hamster monoclonal anti-human GM2 Ab was a kind gift from Dr. Kenneth Rock, UMass, Worcester, MA, Dana Farber Cancer Institute (DFCI) and Corixa Corp., Seattle, WA, USA. RIPA, protease inhibitor and BCA reagents were purchased from Pierce Scientific, USA. HRP-rabbit anti-mouse IgG (2°Ab) was purchased from Cell Signaling Technologies, USA. Plasmid isolation and PCR purification kits were purchased from Qiagen, USA. Alexa-fluor goat anti-hamster IgG was purchased from Molecular Probes, NY, USA. Fibronectin and apoptosis kit were purchased from BD Biosciences, CA, USA. Vectashield mounting media was obtained from Vector Laboratories, CA, USA.

### Cell Culture

Mouse kidney cancer (Renca) cell line was originally provided by Jim Finke's lab (Cleveland Clinic, Ohio). GM2 over-expressing Renca-v and low GM2 expressing (12)1/CA cell lines were cultured in complete RPMI-1640 containing 10% FBS, 1 mM sodium pyruvate, 2 mM L-glutamine, non–essential amino acids, 100 units/ml penicillin, 100μg/ml streptomycin and 50μg/ml gentamycin sulfate. Mouse fibroblast cell line, NIH3T3 was maintained in DMEM supplemented with 4.5 mg/ml glucose and other ingredients stated above.

### Design and assembly of TALEN Pair

Murine GM2-synthase TALEN pairs were designed using the online software ZiFiT Targeter v4.2*.* A genomic region including the translation start site within mouse GM2-synthase locus was run on the online tool “Tale nucleases (REAL and REAL-Fast)” (http://zifit.partners.org/ZiFiT/ChoiceMenu.aspx) to design the TALENs. TALEN pairs were assembled by following the published protocol using REAL method with some modifications^25^. Hierarchical ligation of TALE monomers in a series of 4 steps generated left and right TALE repeats each consisting of 16mers as shown in supplementary figure S1b (step 4). Each 16mer TALE repeats were then cloned into a suitable mammalian expression vector containing a N-terminal FLAG tag followed by N-terminal TALEN start monomer targeting the base 'T' and a C-terminal tagged with “0.5” TALE repeat domain targeting specific base followed by C-terminal tagged with wild-type Fok1 nuclease domain as depicted in supplementary figure S1c. Finally, both left and right TALENs were verified by sequencing using ABI-3500 genetic analyzer. Sequencing primers were listed in supplementary table S1.

### Transfection and assessment of efficacy of the TALEN Pairs

Activity of GM2/GD2-synthase specific TALEN pairs were tested by transfecting Renca-v and NIH3T3 cells with an equal amount of plasmids encoding left and right TALEN. Briefly, 3 × 10^5 ^cells were transfected with 3μg of each TALEN by Lipofectamine-2000. TALEN expression was checked by western immunoblotting by probing with anti-FLAG or anti-β-actin antibody and immuno-detected using HRP conjugated 2°-antibodies (rabbit anti-mouse IgG and goat anti-rabbit IgG). To assess TALEN induced mutation at the GM2/GD2-synthase target site by T7E1 assay, 50 ng of genomic DNA was used to amplify the target region using primer set T7E1F + T7E1R (primers listed in supplementary table S2). 600 ng of the PCR product was denatured at 95°C and slowly renatured by decreasing temperature 0.5°C/30 sec till 20°C to form heteroduplex^26^. The amplicons were digested with 5U of T7E1 enzyme for 45 mins and analyzed by 2% agarose gel electrophoresis.

### Off-target effect analysis

“TAL Effector Nucleotide Targeter, v2.0” (https://tale-nt.cac.cornell.edu/node/add/talef-off-paired) was used to find off-target regions throughout the mouse genome by running left and right RVDs^26^ with the following possible combination of TALEN binding: RVD1 + RVD1, RVD1 + RVD2, RVD2 + RVD1, RVD2 + RVD2. Result from this tool identified huge number of off-target regions in the mouse genome with different TALEN binding score. Low TALEN binding score indicates high specificity of TALEN pair and *vice-versa*. Hence, five potential off-target regions were chosen from different chromosomes given by the program on the basis of low TALEN pair binding score. 72 hr's post-transfection, target regions were PCR amplified (primers listed in supplementary table S3) from wild-type and TALEN pair transfected cells and digested with T7E1 as described before.

### Isolation of complete and stable GM2-synthase targeted cells using clonal selection

For this, we used a co-transfection procedure described before^27^. 5 × 10^4^ cells were transfected with 400 ng each TALENs (L + R) and 10 ng of pDsRed-ExpressC1 empty vector (containing a neomycin resistance cassette) (40:40:1 ratio). 48 hr's post-transfection, cells were selected against 1μg/ml G418 to form colonies from a single cell and further expanded in 24-well plates. Either genomic DNA was isolated to perform PCR genotyping or grown on coverslips to perform immuno-staining for assessing GM2 expression. Clones having no GM2 expression were used for further experiments.

### Immunofluorescent staining

GM2 expression in 24 clones was checked by immunofluorescent microscopy by methods described previously^53^. Briefly, 5 × 10^3 ^cells grown on coverslips were fixed with 3.7% paraformaldehyde, washed with 1X PBS and stained with hamster anti-GM2 antibody (1:50) overnight at 4°C. Cells were washed with PBS, counterstained with Alexaflour-488 goat anti-hamster IgG antibody (1:500) for 1 hr at room temperature. Cells were washed with PBS, mounted on slides and observed using Leica fluorescence microscope.

### PCR Genotyping

To assess TALEN induced mutation of GM2-synthase target region and to determine mutation rate, PCR genotyping was performed by methods discussed elsewhere^22^. 50 ng of genomic DNA isolated by phenol-chloroform method from all 24 clones was applied for PCR using primer set GM2SF + GM2SR (primers listed in supplementary table S4), yielding 101 bp amplicon encompassing TALEN target region. PCR products were analyzed by 4% high resolution agarose gel to resolve and identify mutations by band shifting.

### TA cloning and sequencing

To identify the ‘indels’ at TALEN target region, PCR reaction was performed to amplify the TALEN target region (primers listed in supplementary table S5). The PCR products were gel eluted, cloned into pTZ57R/T and sequenced to confirm mutation in the target region using ABI-3500 genetic analyzer.

### Construction and transfection of donor plasmid

The 101 bp bait sequence of GM2-synthase TALEN target region was PCR amplified from Renca-v genomic DNA using primer set (F1 + R1). PCR product was cloned into pTZ57R/T and then digested with Xba1 and BamH1. Separately, CMV-neomycin cassette, amplified from empty pDsRed-Express C1 using Apa1 restriction site tagged at the forward and reverse primer was cloned into pTZ57R/T at Apa1 site. This pTZ57R/T-CMV-Neomycin vector was then digested with Xba1 and BamH1 and used to clone the 101 bp bait sequence to generate the donor vector containing the bait sequence and neomycin cassette (Fig. 4b). 3 × 10^5 ^cells were then transfected with either 1.2μg of each TALEN and 2μg and 3μg of donor plasmid or 4μg of donor plasmid alone. To check NHEJ-mediated integration of the CMV-neomycin cassette^28^, genomic DNA was extracted and PCR was performed. PCR primers for donor plasmid construction and assessment of NHEJ-mediated integration were listed in supplementary tables S6 and S7.

### MTT proliferation assay and cell counting to assess cell viability

Viability of complete GM2 knockout cells was measured either by MTT proliferation assay^34^ or cell counting. Briefly, 24 hr's, 48 hr's and 72 hr's post-seeding (4 × 10^4^ cells/well), MTT (0.5 mg/ml) was added. 3 hr's after incubation at 37°C, cells were washed with PBS, 1 ml of DMSO was added and absorbance was measured at 570 nm. Cell counting was performed following trypsinization using hemocytometer at the mentioned time points (4 × 10^3^ cells/well).

### RNA isolation and real-time PCR

Total RNA was extracted using Trizol reagent (Invitrogen). cDNA was prepared from 1μg of total RNA using verso cDNA synthesis kit (Thermo Scientific). Real-time PCR was performed in triplicate with 2μl of a diluted (1:5) stock of cDNA using SYBR green PCR system on 7500 Fast (Applied Biosystems). All mRNA quantification data were normalized to GAPDH and expressed as the fold differences of target gene expression relative to the wild type. Real-time primers were listed in supplementary table S8.

### Soft agar colony formation assay

For soft agar assay^2^, 2 × 10^3 ^wild type and knockout cells were resuspended in 1 ml 2X complete RPMI-1640 and mixed with 1 ml warmed 0.7% agarose and plated over a solidified 0.75% agarose in 1X RPMI-1640. Following solidification of the top layer, 2 ml of RPMI-1640 was added, incubated at 37°C for 15 days. Colonies were fixed, stained with Giemsa and imaged using Gel Doc XR + (Bio-Rad).

### Effect of GM2-synthase knockout in primary tumor model

Renca-v wild type and Renca-v^GM2-synthase−/−^ (KO-2) mouse tumor cells were used to develop tumors in a well established syngeneic tumor model using Balb/c mice^36^,^37^. Briefly, 3 × 10^6^ wild type as well as Renca-v^GM2-synthase−/−^ (KO-2) cells were injected into healthy mice to establish intradermal tumors and allowed to grow for 31d post injection. Development of tumors in mice injected with knockout cells were compared with wild type, by measuring both tumor size and also the rate at which tumor develops. Perpendicular bi-dimensional measurements were performed every three days and tumor volume was expressed as the size (cm^3^) of bi-dimensional product. All animal experiments were conducted in accordance with strict guidelines and all experimental protocols have been approved by the institutional animal ethics committee of CSIR-IICB, registered with the Committee for the Purpose of Control and Supervision of Experiments on Animals (CPCSEA), India (Permit 147/1999/CPCSEA).

### Clonogenicity and cell adhesion assay

For clonogenicity assay^17^, cells were plated at very low density (200 cells/60 mm dish), grown for either 5/7 days, fixed with 3.7% formaldehyde, stained with 0.05% crystal violet and image was captured using Gel Doc XR + (Bio-Rad). 10X magnified images of individual clones were captured by Leica microscope. For cell adhesion assay^38^, 2.5 × 10^4 ^cells were plated in each well of 24 well culture plates coated with 10μg/ml fibronectin and incubated at 37°C for 15 mins. Plates were washed with PBS, fixed with 3.7% formaldehyde, stained with 0.05% crystal violet and images of cells were taken in 10 × magnification using Leica microscope.Then 2% SDS was added, incubated for 30 mins and absorbance was measured at 570 nm.

### Anoikis Assay

To determine detachment induced cell death, 4 × 10^5^cells were plated on LAPs in RPMI-1640 with 1.25% methyl cellulose^52^. After 24 hr's, cells were washed twice with PBS and % cellular death was measured by acquiring PI/Annexin-V positive cells in FACS (BD FACS Verse) using Apoptosis Detection Kit as per manufacturer's instruction.

### Statistical Analysis

Statistical analysis was performed by one-way ANOVA followed by Dunnett's multiple comparison tests using Graphpad Prism 5.0. For tumor volume calculations and real-time PCR data analysis “paired t-test” has been used. Values were considered as statistically significant when p value was less than 0.05.

## Author Contributions

K.B. supervised the entire work presented in this manuscript. B.M. performed most of the experiments presented in the manuscript. A.B. and M.K. performed real-time PCR for analysis of EMT markers. Both B.M. and K.B. prepared figures and wrote the manuscript text. U.B. provided intellectual inputs in preparation of the manuscript and help with the animal experimentation. All authors reviewed the manuscript.

## Supplementary Material

Supplementary Information
