# Supplementary material for: TALEN mediated targeted editing of GM2/GD2-synthase gene modulates anchorage independent growth by reducing anoikis resistance in mouse tumor cells
Source: Sci Rep. 2015 Mar 12;5:9048. doi: 10.1038/srep09048 (PMC4357006; doi:10.1038/srep09048)

Supplementary Information for

TALEN mediated targeted editing of GM2/GD2-synthase gene modulates anchorage independent growth by reducing anoikis resistance in mouse tumor cells

Barun Mahata[1], Avisek Banerjee[1], Manjari Kundu[1], Uday Bandyopadhyay[2] and Kaushik Biswas[1]*

[1]Division of Molecular Medicine, Bose Institute, Kolkata, India, [2]Department of Infectious Diseases and Immunology, CSIR-Indian Institute of Chemical Biology, Kolkata, India

* To whom all correspondence should be addressed. Correspondence should be addressed to Kaushik Biswas, P1/12 CIT Scheme VIIM, Division of Molecular Medicine, Bose Institute, Kolkata-700054, India, Email : kaushik@jcbose.ac.in

Supplementary Figures

Figure S1 Related to Figure 1

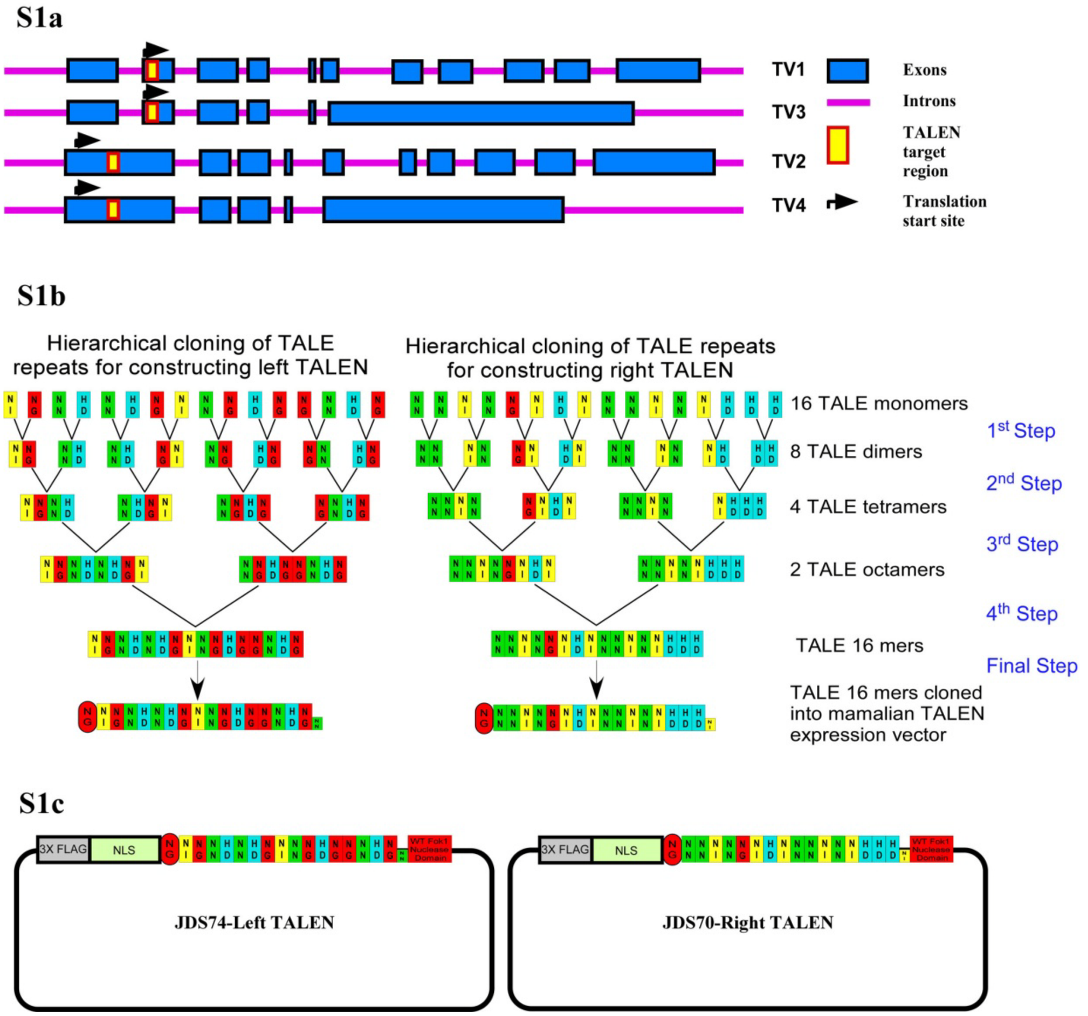

Figure S1. Strategy and vector map of murine GM2-synthase specific TALEN pair. *Fig. S1a* represents all four transcript variants (TV1, TV2, TV3 and TV4) of mouse GM2-synthase gene. Blue boxes indicate exons, purple line indicate adjacent introns, yellow box with red outline indicates TALEN target region and black arrows indicate translation start site. *Fig. SIb* represents construction of left and right TALEN using REAL strategy. First step includes cloning of two TALE monomers, 2nd step includes cloning of two TALE dimers, 3rd step includes cloning of 2 TALE tetramers, 4th step includes cloning of 2 TALE octamers to generate TALE 16 mers for both left and right TALEN. In the final step, each TALE 16 mers were then cloned into mammalian TALEN expression vector, a N-terminally tagged with FLAG and a TALEN starting TALE monomer targeting base T and C-terminally tagged with “0.5” TALE repeat domain with 20 amino acids and wild type Fok1 nuclease domain. *Fig. S1c* shows schematic representation of left and right TALEN expression plasmids**.** Grey box and light green box N-terminal to TALEN repeat modules represents the 3X FLAG and nuclear localizing (NLS) sequence respectively. Wild type Fok1 nuclease domain (red box) is tagged at the C-terminal end of TALEN module. Ligation strategy of TALE repeats modules were described in results.

Figure S2 Related to Figure 3

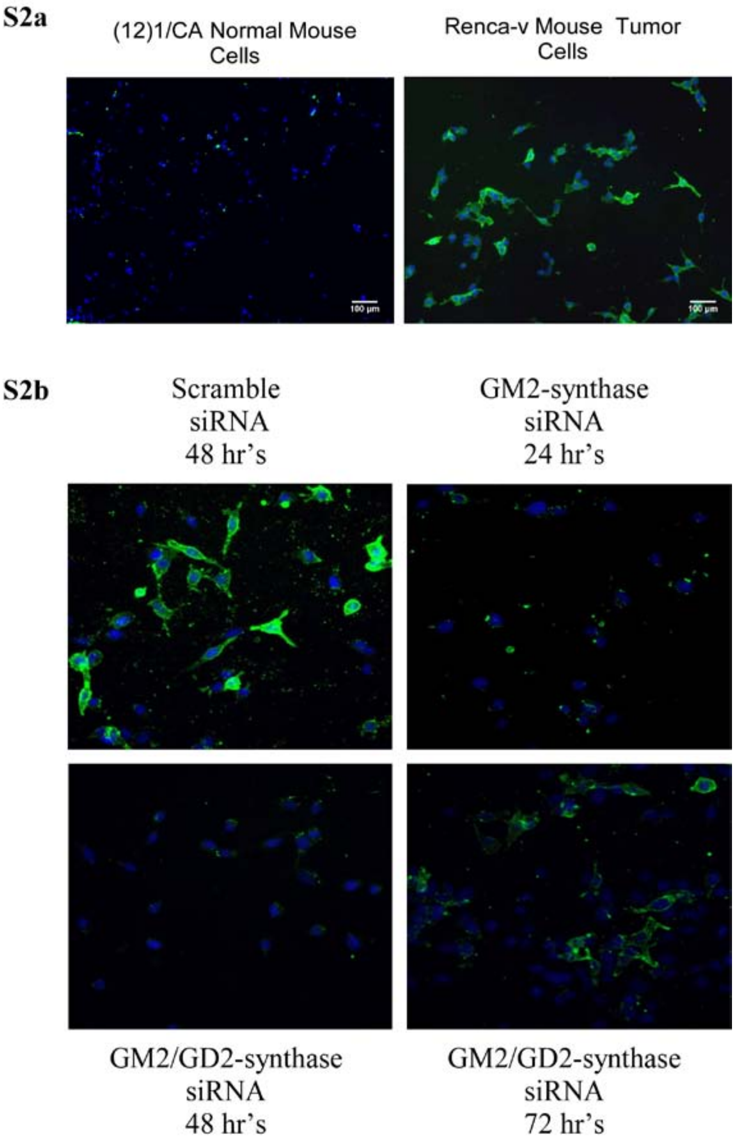

Figure S2. Transfection of GM2/GD2-synthase siRNA shows time-dependent GM2 knockdown in Renca-v cells. *Fig. S2a* shows expression profile of ganglioside GM2 in normal mouse fibroblast (12)1/CA and mouse tumor Renca-v cells. *Fig. S2b* shows siRNA mediated downregulation of GM2 expression and reversion of GM2 expression. In brief, 1 day before transfection, 1.5 x 10^5 cells were plated in 6 well plates in antibiotic free complete RPMI-1640. Transfection was done by 150 picomole siRNA (GM2/GD2-synthase or scramble) using 5μl of Lipofectamine-2000. 12 hr's post-transfection, 5 x 10^4 cells were plated on coverslip in 12-well format. After a total of 24 hr's, 48 hr's and 72 hr's post-transfection, cells were washed, fixed and immunostained with hamster anti-human GM2 Ab (1ºAb) at 4ºC overnight. Cells were counterstained with FITC conjugated anti-hamster (2ºAb) and then mounted on a slide using vectashield mounting media. The slides were then visualized under a fluorescent microscope, (Leica, DM IL LED). Green fluorescence indicates the GM2 expression while blue fluorescence indicates nucleus of the cells stained with DAPI.

Figure S3 Related to Figure 5e

S3

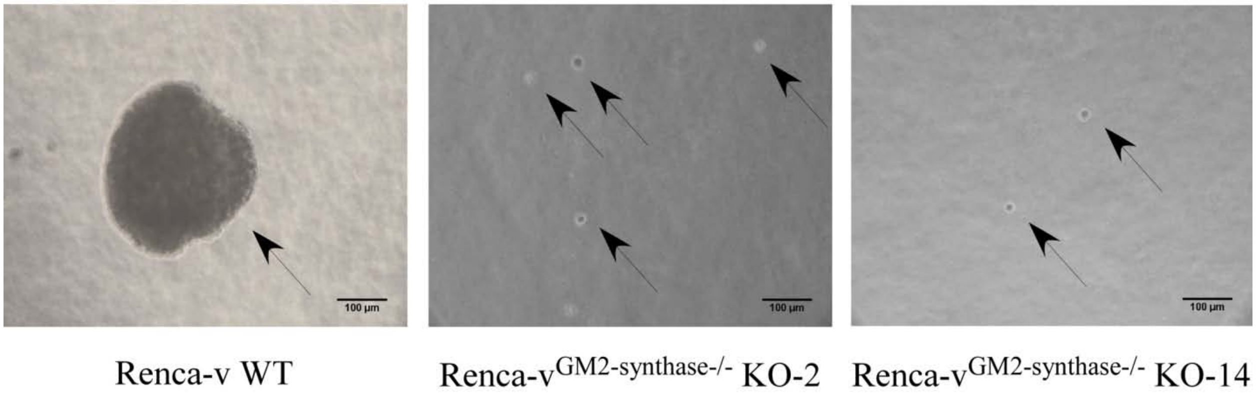

Figure S3. TALEN mediated disruption of GM2-synthase resulted in significant reduction of colony size in Renca-v cells. *Fig. S3* shows representative phase contrast micrograph images (10X magnifications) of colonies from wild type and two GM2-synthase KO clones taken by Leica, DM IL LED microscope. Briefly, $2{\times}10^3$ cells were resuspended in 1ml 2X RPMI-1640 and mixed with 1ml warmed 0.7% agarose so that final concentration of RPMI was 1X and agarose was 0.35% and plated over a solidified 0.75% agarose in 1X RPMI-1640. Following solidification of the top layer, 2ml of RPMI-1640 was added. Plates were incubated in humidified cell culture incubator supplied with 5% CO_2 for 15 days. Media was changed every three days.

Figure S4 Related to Figure 6d

S4

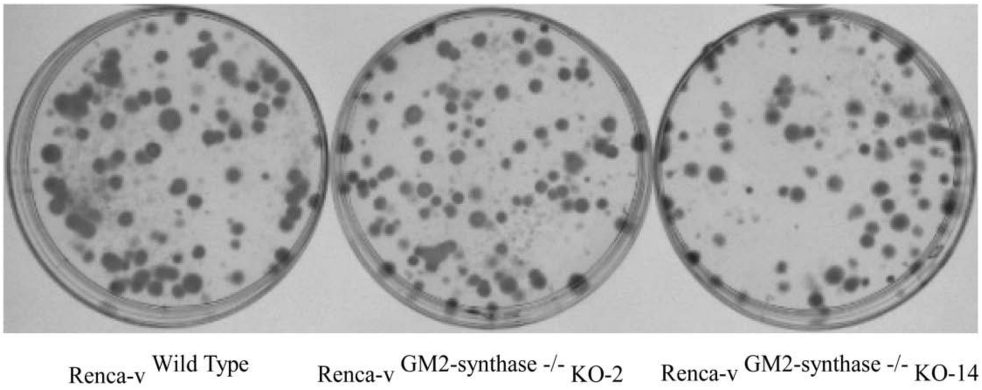

Figure. S4. TALEN mediated knockout of GM2-synthase have no effect on clonogenicity of Renca-v cells. Briefly, very low number (200) of cells were plated in 60mm dish in complete RPMI-1640 and grown for 7 days. The colonies were then fixed with 3.7% paraformaldehyde, stained with 0.05% crystal violet and image was captured using Gel Doc XR+ (Bio-Rad).

Supplementary Tables

Table S1. Primers used to verify the sequence of TALEN pair

Primer Name	Primer Sequence (5'-3')	Primer Description
**OK 163**	CGCCAGGGTTTTCCCAGTCACGAC	Used to sequence intermediate TALE repeats
**JDS 2978**	TTGAGGCGCTGCTGACTG	Forward primer used to sequence verify TALEN in expression vector
**JDS 2980**	TTAATTCAATATATTCATGAGGCAC	Reverse primer used to sequence verify TALEN in expression vector
**JDS 2778**	CTGGCGCAATGCGCTCAC	Forward sequencing primer closer to TALE repeat region
**JDS 2979**	AAGCAATGGCGACCACCTGTTC	Reverse sequencing primer closer to TALE repeat region

Table S2. Primers used to access TALEN mediated cleavage activity at GM2-synthase locus

Primer Name	Primer Sequence (5'-3')	Primer Description
**T7E1F**	CGCACCCAAGATACTGCATGTCACC	Forward primer used for T7E1 assay
**T7E1R**	AAGCTTCGGTAGCCCTCTCCACCTC	Reverse primer used for T7E1 assay

Table S3. Primers used to analyse the off-target effect mediated by GM2-synthase TALEN pair

Primer Name	Primer Sequence (5'-3')
**Chr. 9 Forward**	CCTCTGTGGTCATATGGATTGTGC
**Chr. 9 Reverse**	TGAGTATAGAAAAAGGTCCTTAGG
**Chr. 10 Forward**	GAAAGGTAATATACACATGAATGA
**Chr. 10 Reverse**	CTAACACCTTCTTTATCTTCTGAG
**Chr. 13 (1) Forward**	TAAGTTTGCAAGACTTTAGTGGGC
**Chr. 13 (1) Reverse**	TGCTGAGTGTACAATTGGGAGGAT
**Chr. 13 (2) Forward**	TTCACCAAGGAAACAGAGCCCTGC
**Chr. 13 (2) Reverse**	TGCTCTGGAGGTGAGTAGAATCAG
**Chr. 15 Forward**	AGACTGTGGGGGTAGAGGGGAAGA
**Chr. 15 Reverse**	TGGGCCCTCCTTATTACTTAGTTA

Table S4. Primers used for PCR genotyping to estimate TALEN induced mutation frequency in isolated clone

Primer Name	Primer Sequence (5'-3')	Primer Description
**GM2SF**	ATGCGGCTAGACCGCCGGGCCCTCTA	Forward primer used for PCR genotyping
**GM2SR**	GGGAGACTTGGCGCGTTTCGGGTGCT	Reverse primer used for PCR genotyping

Table S5. Primers used to clone GM2-synthase TALEN target region as well as for sequencing to assess indel mutations

Primer Name	Primer Sequence (5'-3')	Primer Description
GM2-synthase TA cloning Forward	CGGAAGAAAGGAGGCCGGGAGACC	Forward primer ~210bp upstream TALEN target region
GM2-synthase TA cloning Reverse	CGCCACGCCGCGGTCCGCACTCAC	Reverse primer ~210bp downstream of TALEN target region
T7 Forward Primer	TAATACGACTCACTATAGGG	Uused for sequencing TALEN induced indels cloned into pTZ57R/T vector
M13/PUC sequencing primer	GTAAAACGACGGCCAGT	Used for sequencing TALEN induced indels cloned into pTZ57R/T vector

Table S6. Primers used to construct donor vector for integration of neomycin cassette into GM2-synthase TALEN target region

Primer Name	Primer Sequence (5'-3')	Primer Description
**F1**	ATGCGGCTAGACCGCCGGGCCCTCTA	Forward primer for bait sequence
**R1**	GGGAGACTTGGCGCGTTTCGGGTGCT	Reverse primer for bait sequence
**F4**	TACGGGCCCTTTCTAAATACATTCAAATATGTAT	Apa 1 tagged neomycin cassette forward primer
**R4**	TACGGGCCCGCGTTTATGAACAAACGACCCA	Apa 1 tagged neomycin cassette reverse primer

Table S7. Primers used to check NHEJ mediated integration of neomycin into GM2-synthase locus

Primer Name	Primer Sequence (5'-3')	Primer Description
**F2**	TTGTGAATCCAAGGGAGGAAGCCT	Forward primer beyond GM2 synthase TALEN target region
**R2**	GTTGCAGCTGCCGGTTGAGTTTAT	Reverse primer beyond GM2 synthase TALEN target region
**F3**	CGGAAGAAAGGAGGCCGGGAGACC	Forward primer used to assess NHEJ mediated integration event
**F4**	TACGGGCCCTTTCTAAATACATTCAAATATGTAT	Apa 1 tagged neomycin cassette forward primer
**R4**	TACGGGCCCGCGTTTATGAACAAACGACCCA	Apa 1 tagged neomycin cassette reverse primer (reverse primer used to assess NHEJ mediated integration event)

Table S8. Primers used for real-time PCR

Primer Name	Primer Sequence (5'-3')
KLF6 Forward	CACACCCACACACACATACA
KLF6 Reverse	ACAGATAGCTAGACAGGTACTCAA
Col3A1 Forward	CCGAACTCAAGAGTGGAGAATAC
Col3A1 Reverse	AATCTGTCCACCAGTGCTTAC
Zeb1 Forward	TACTTGCCTCCGACTGTAGA
Zeb1 Reverse	TGGCTTGCTAAGGGAATGAG
ADAM10 Forward	TGCCTTGGAATTATGTTGGAAATG
ADAM10 Reverse	GATCAGTGACAGTGCTGAAGAA
ADAM 9 Forward	GAACAGGAGCAGGGAGAATAC
ADAM 9 Reverse	GACCCAGCACACCTTAGTAAAT
β- Catenin Forward	CCCTAGCCTTGCTTGTTCTT
β- Catenin Reverse	GTTCTACACCATTACTCGGTTCT
Vimentin Forward	CAAGCAGGAGTCAAACGAGTA

Vimentin Reverse	GCCAATAGTGTCCTGGTAGTTAG
GAPDH Forward	GGAGAAACCTGCCAAGTATGA
GAPDH Reverse	CCAGGAAATGAGCTTGACAAAG

Original GEL Slides

Figure 1b FLAG TAGGED TALEN

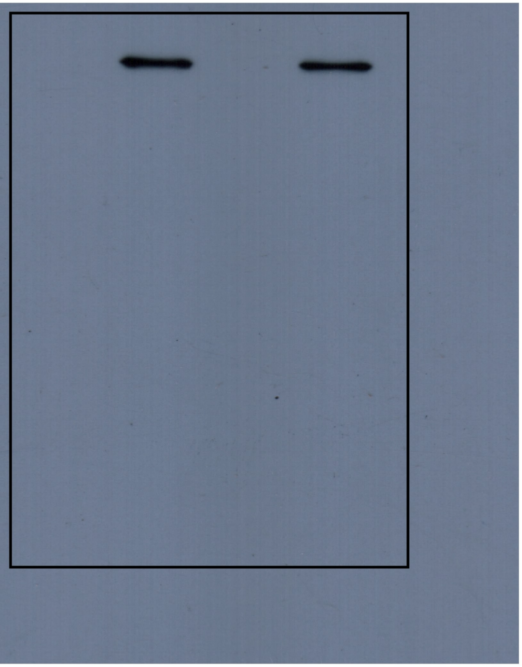

Figure 1b beta ACTIN

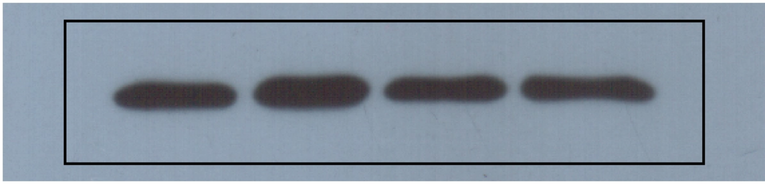

Figure 1d T7E1 ASSAY

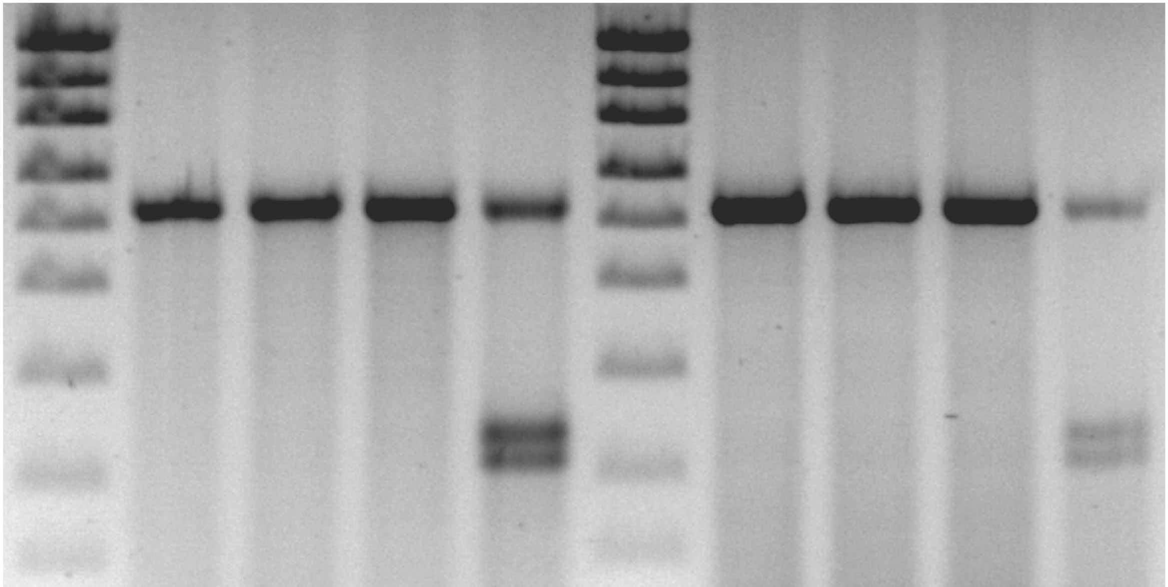

Figure 2b   OFF TARGET ANALYSIS by T7E1 ASSAY

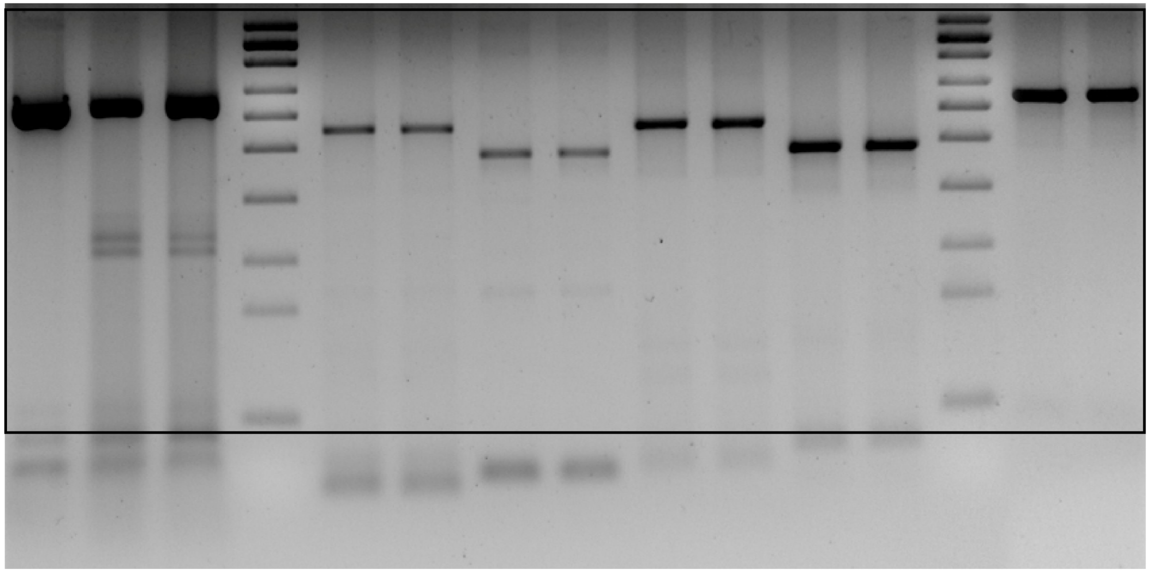

Figure 3b   PCR GENOTYPING CLONES 1-12

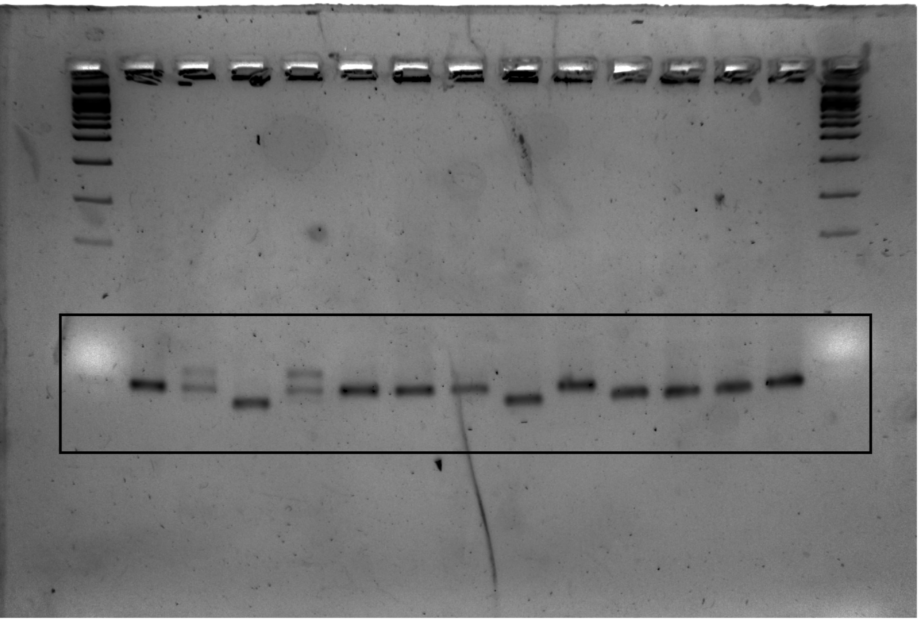

Figure 3b   PCR GENOTYPING CLONES 13-24

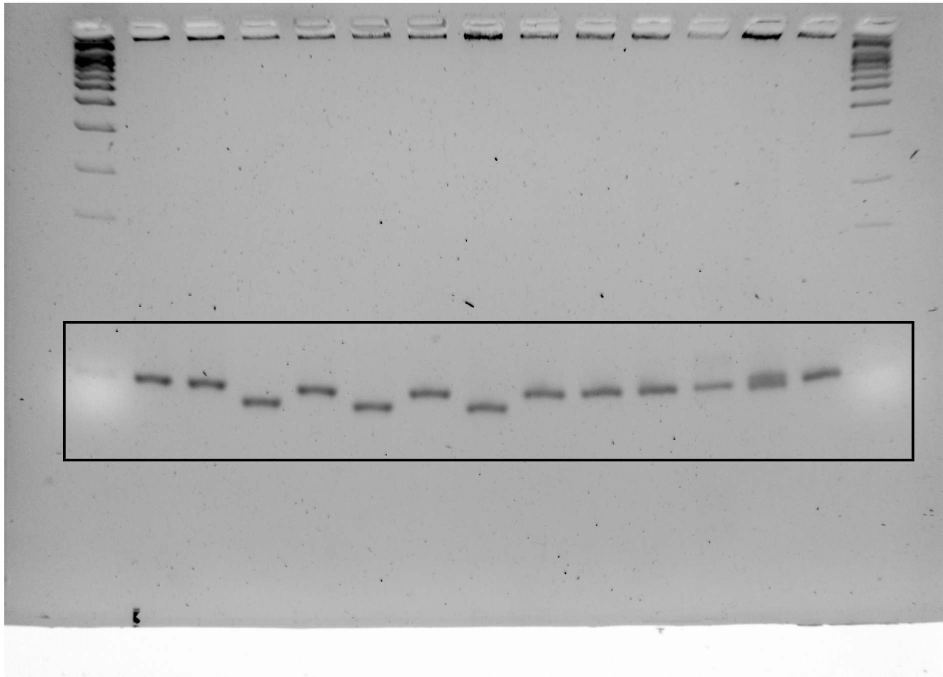

NHEJ MEDIATED INTEGRATION OF NEOMYCIN CASSETTE

FIGURE 4d (F3+R4)

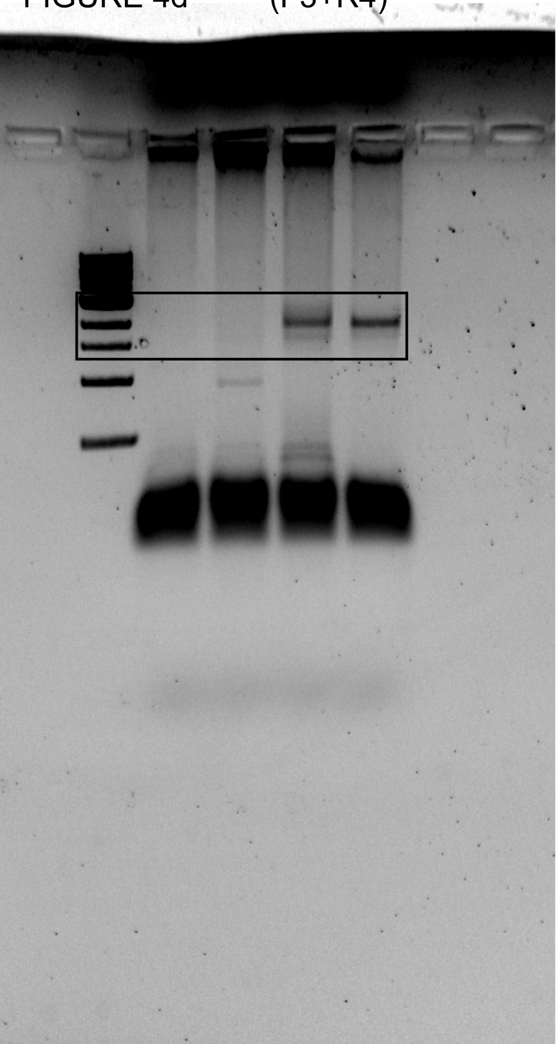

FIGURE 4d (F4+R4)

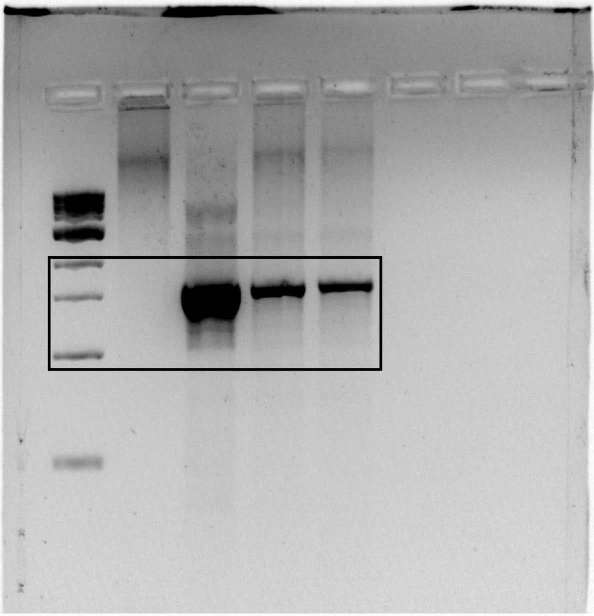

FIGURE 4d (F2+R2)

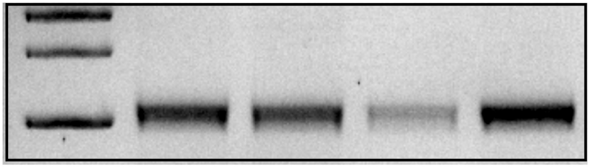

Supplement: Supplementary Information [file srep09048-s1.pdf]
